# Characterization of Acute and Chronic Hepatitis B Virus Genotypes in Canada

**DOI:** 10.1371/journal.pone.0136074

**Published:** 2015-09-25

**Authors:** Carla Osiowy, Elizabeth Giles, Max Trubnikov, Yogesh Choudhri, Anton Andonov

**Affiliations:** 1 National Microbiology Laboratory, Public Health Agency of Canada, Winnipeg, Manitoba, Canada; 2 Centre for Communicable Diseases and Infection Control, Public Health Agency of Canada, Ottawa, Ontario, Canada; Centers for Disease Control and Prevention, UNITED STATES

## Abstract

**Objective:**

The prevalence and distribution of hepatitis B virus (HBV) genotypes in Canada is not known. Genotypic analysis may contribute to a better understanding of HBV strain distribution and transmission risk.

**Methods:**

HBV surface antigen (HBsAg) positive samples of acute (n = 152) and chronic (n = 1533) HBV submitted for strain analysis or reference genotype testing between 2006 and 2012 were analyzed. The HBsAg coding region was amplified to determine the HBV genotype by INNO-LiPA assay or sequence analysis. Single and multivariate analyses were used to describe genotypes’ associations with known demographic and behavioral risk factors for 126 linked cases of acute HBV.

**Results:**

Nine genotypes were detected (A to I), including mixed infections. Genotype C (HBV/C) dominated within chronic infections while HBV/D and A prevailed among acute HBV cases. History of incarceration and residing with a chronic HBV carrier or injection drug user were the most frequently reported risks for acute HBV infection. Over time, HBV/A increased among both acute and chronic infections, and HBV/C and HBV/D decreased among chronic infections.

**Conclusion:**

Chronic and acute HBV genotypes in Canada differ in the relative distribution and their associations with known risk factors, suggesting different routes of transmission and clinical progression of infection.

## Introduction

The prevalence of chronic hepatitis B virus (HBV) infection in Canada has been estimated at <1% [1, 2]; however, the HBV burden among specific populations, such as injection drug users (IDU) or immigrants from countries where HBV is endemic, is considerably higher (≥ 2%-10%; [1, 3–5]). In 2011, immigrants comprised almost 21% of the total population of Canada [6], with the majority of individuals originating from countries with intermediate (2–7%) or high (≥8%) HBsAg seroprevalence [7]. Acute cases of HBV infection in Canada are decreasing, with an incidence rate declining from 0.97 to 0.49 per 100,000 inhabitants between 2005 and 2010, according to the findings of the former Enhanced Hepatitis Strain Surveillance System (EHSSS), which ran from approximately 1998 to 2012 [8]. This reduction is believed to be due to implementation of HBV vaccination programs, improvements in infection control and public health measures, and enhanced detection of transfusion transmitted viruses [1, 9].

The 8 major genotypes of HBV, A to H, have a distinct geographic distribution throughout the world [10], and evidence has accumulated to show an association of genotypes with long-term outcomes or response to interferon therapy [11]. The National Microbiology Laboratory performs HBV reference diagnostic service for the Provinces and Territories of Canada, including genotype and mutation analysis. Thus, in order to determine the distribution of HBV genotypes circulating in Canada, HBV DNA positive referral and EHSSS specimens received between 2006 and 2012 were investigated. Here we report for the first time the proportion and distribution of HBV genotypes throughout Canada, associated with chronic and acute infection.

## Methods

### Study populations and design

The National Microbiology Laboratory (NML) is the national reference laboratory for diagnostic testing and surveillance of viral hepatitis in Canada. Specimens are routinely received for HBV genotyping and represent patients newly referred to specialists, candidates for treatment, or targeted populations, such as HBV-infected pregnant women. Thus all samples were assumed to be from chronically-infected hepatitis B patients and so represent the HBV prevalent population in Canada. Only age and gender were available for the majority of reference requests. Reference requests submitted to the NML from 2006 to 2012 from 9 Provinces and 1 Territory, representing Western, Prairie, Central and Maritimes regions of Canada, were included in the study (n = 1533). The number of specimens collected and analyzed each year from 2006 to 2012, respectively, was 79, 108, 214, 82, 297, 389, and 364.

Specimens (n = 152) were received at the NML for EHSSS laboratory strain surveillance of acute HBV from participating sites within 3 Provinces, representing Western and Central regions of Canada, and diagnosed between 2006 and 2012, with the following annual breakdown of the analyzed specimens: 30, 19, 26, 32, 24, 15 and 6. Linkage between genotype and EHSSS case records providing demographic and behavioral risk factor information was successful for 126 specimens. The case definition for acute HBV infection was previously described elsewhere [12] and included a discrete onset of clinical symptoms (abdominal pain, jaundice, fatigue, nausea, etc.) along with serum aminotransferase levels > 2.5 times the upper limit of normal, and serological verification of acute infection (HBV surface antigen (HBsAg) positive, antibody to HBV core protein (anti-HBc) IgM positive, anti-hepatitis A virus IgM negative). Confirmation of each case required a detailed patient clinical history and/or evidence of exposure compatible with acute HBV infection. Informed consent was obtained from each participant for the collection of a serum specimen and epidemiological data providing demographic and risk factor information. The risk factors specifically analyzed in the present study included sex, age, race, reported history of immigration, piercing or tattoo, occupational exposure to high-risk biological substances, having resided with an HBV carrier or IDU, history of invasive medical procedures, such as surgeries, dental surgeries, endoscopies, and repeated injections/blood drawing due to chronic health conditions, history of repeated testing for sexually transmitted and blood-borne infections (STBBI), history of HBV vaccination, history of IDU, non-injection drug use, unprotected sexual intercourse with a person known to have HBV infection, and life-time same-sex sexual intercourse, and the history of incarceration. Presence of clinical symptoms specific and non-specific to the liver disease and a range of serological measures, including results for HBV e antigen (HBeAg) test, alanine aminotransferase (ALT) test, antibody to hepatitis C virus (HCV) and HCV-RNA tests were also analysed. The Health Canada Research Ethics Board approved the EHSSS study protocol and consent procedure. At the time of recruitment, participants were provided with a printed copy of the study description, including contact information for principal investigators. Following a verbal explanation of the study purpose and the rights of participants, individuals were asked if they agree to participate in the study, and if positive, written consent was obtained at that time. Consenting participants signed a consent statement within the printed study description and interviewers populated a participant response category within the epidemiological data questionnaire. Hard copies of consent statements and filled-in questionnaire data were securely stored by EHSSS site investigators and paper-based data was transferred to an electronic format of an Access database maintained by the EHSSS site. This process was followed by all participating sites until 2010, when an on-line data entry system, securely maintained by the Public Health Agency of Canada, was devised that ensured a more streamlined data transfer process between EHSSS sites. All research was conducted according to the principles expressed in the Declaration of Helsinki.

Raw data for acute and prevalent cohorts are available as supplementary data (S1 and S2 Tables).

### HBV DNA extraction and amplification

DNA was extracted from 200 μl of serum as previously described [13] or by silica capture and stored at -20°C. The partial HBsAg genomic region was amplified using AmpliTaq Gold DNA Polymerase (Life Technologies, Mississauga, Ontario, Canada) according to the manufacturer’s protocol, using primers and conditions as described previously [14], to obtain a product of approximately 343 bp. Alternatively, amplification and sequencing was performed using primers HBVsF01in (5' ACCCTGYRCCGAACATGGA 3') and DRv2as (5' AGAAAGGCCTT-GTAAGTTGGCGA 3') to obtain a product of approximately 980 bp. Thermal cycling parameters involved 35 cycles of 94°C for 30 s, 55°C for 30 s, and 72°C for 40 s. If necessary, nested PCR was performed using primers DRv2s (5' CGTGG-TGGACTTCTCTCAATTTTC 3') and HBVR743as (5' CAACTCCCAATTACATARCCCA 3') to obtain a product of approximately 644 bp. Thermal cycling conditions were the same as the first-stage reaction, except with 30 cycles.

Extracted DNA from acute case samples was also amplified and sequenced using primers specific for the precore region, according to Takahashi K, et al. [15] to investigate the presence of basal core promoter and precore (BCP/PC) mutations. For all PCR amplifications, strict procedures involving negative controls and spatial isolation of individual reaction steps were followed to prevent environmental contamination.

### HBV sequencing and genotype determination

Genotype determination occurred through HBsAg sequence analysis exclusively for acute case samples and a mixture of INNO-LiPA HBV Genotyping assay (Fujirebio Europe, Gent, Belgium) analysis and sequencing, in cases of LiPA PCR negativity, indeterminate results, or targeted sequence investigation, for prevalent case samples.

Amplicons were gel-purified prior to cycle sequencing using an ABI 3730XL DNA Analyzer (Applied Biosystems, USA). Sequences were assembled and analyzed using Lasergene sequence analysis software (DNASTAR Inc., Madison, WI, USA). The sequence-based HBV genotype was initially estimated using the NCBI HBV Genotyping tool [16] and BLAST analysis. Sequences were aligned and trimmed using ClustalX v2.0.10 [17] and BioEdit v7.0.9 [18], respectively, followed by phylogenetic analysis if sufficient sequence data was available. Maximum likelihood analysis of the partial HBsAg coding-region (trimmed to approximately 519 bp for all sequences, from nucleotide 311 to 829) was performed by the K2+γ+I model (γ: 0.7832 for acute sequences, 0.6537 for chronic sequences; I: 0.5985 for acute sequences, 0.5354 for chronic sequences), while the K2+γ model (γ = 0.2624) was used for analyzing mixed acute and chronic sequences for genotypes A and D only, using DIVEIN (PhyML 3.0) and MEGA5.2 software following model estimation analysis of alignments by MEGA5.2 [19]. Phylogenetic trees were constructed by the BioNJ algorithm [20] with tree topology evaluated by 100 bootstrap replicates. The GenBank accession numbers for prevalent and acute case sequences analysed are KT370116 to KT370859.

HBV genotype determination of reference diagnostic samples by the INNO-LiPA HBV Genotyping assay involved amplification of extracted DNA using assay-supplied, HBsAg-coding region specific, biotinylated primers in a nested PCR reaction, according to the manufacturer’s protocol. Amplified material was denatured and hybridized to genotype specific oligonucleotide probes immobilized on membrane strips, using the AutoBlot3000H (MedTec, Inc. Hillsborough, North Carolina, USA). Following stringent washing and incubation with a streptavidin conjugate, a chromogenic reaction results, indicating hybridization between biotinylated amplicon and genotype-specific probe(s).

### Statistical analysis

Univariate analyses were used to describe the distribution of hepatitis B genotypes and known risk factors. Pearson chi-square, Fisher’s exact test and single-factor logistic regression models were used to test the significance level of associations. Difference in mean age between acute and prevalent cases was analysed by Student’s t-test. To check for the collinearity effect between exposure variables, a correlation matrix of all possible exposures was built and assessed. Logistic regression models were fitted for genotypes found to have significant associations in univariate analyses at p<0.1. Multivariate analysis was conducted by entering all variables found to be significantly associated with specific genotypes. Pearson’ and Hosmer-Lemeshow’ goodness of fit tests were used to assess the effect of removing individual variables. Possible interactions between remaining variables were assessed to determine if these have an effect on the measurement of regression coefficient. Final models were chosen based on likelihood ratio tests that compared models with and without specific variables. All analyses were conducted using STATA 13 (Stata Corporation, College Station, TX, USA) and MS Excel.

## Results

### Genotype distribution

**Fig 1:**
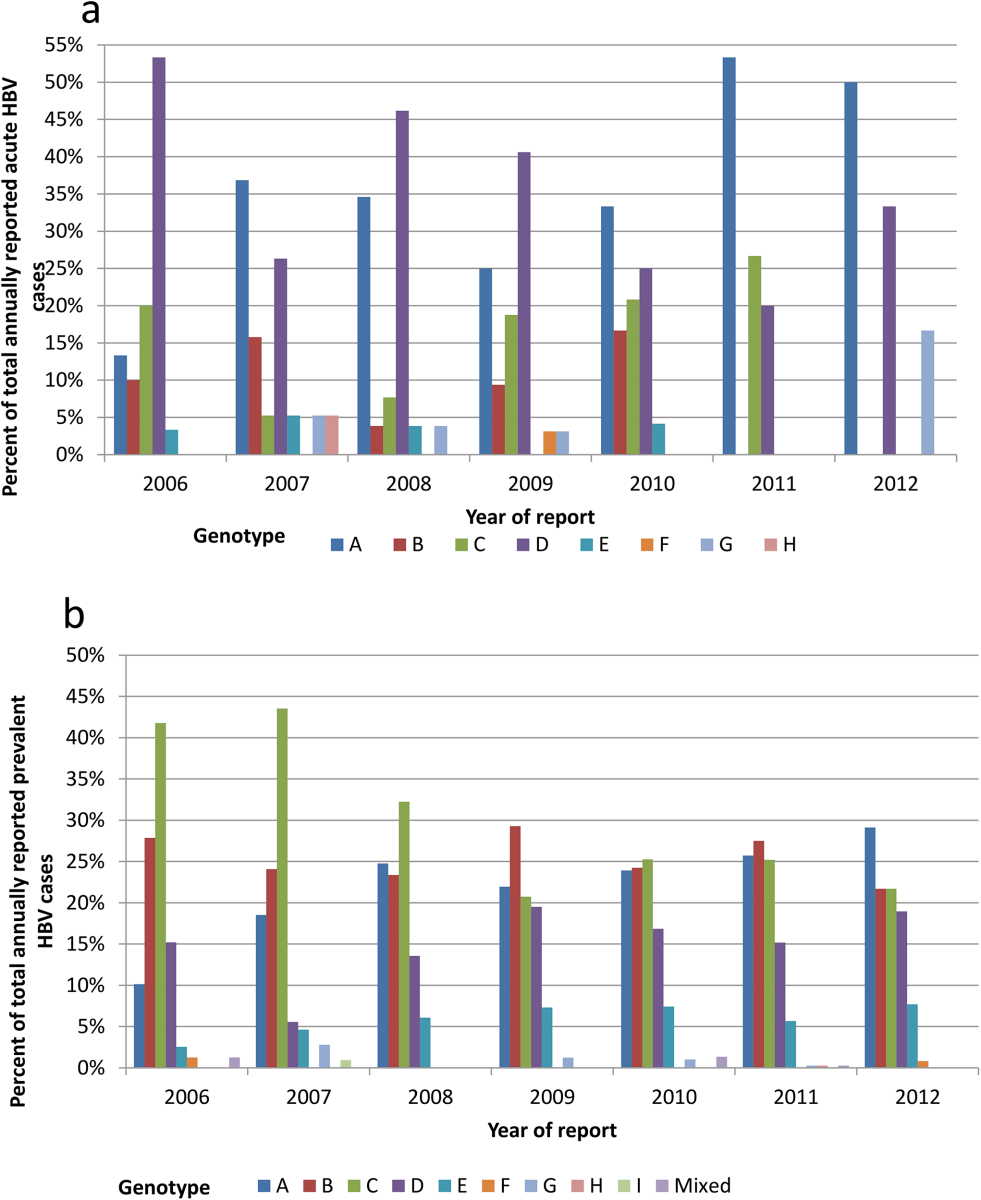
Relative distribution of acute HBV cases by genotype and year of report (a), and relative distribution of prevalent HBV cases by genotype and year of report (b).

**Fig 2:**
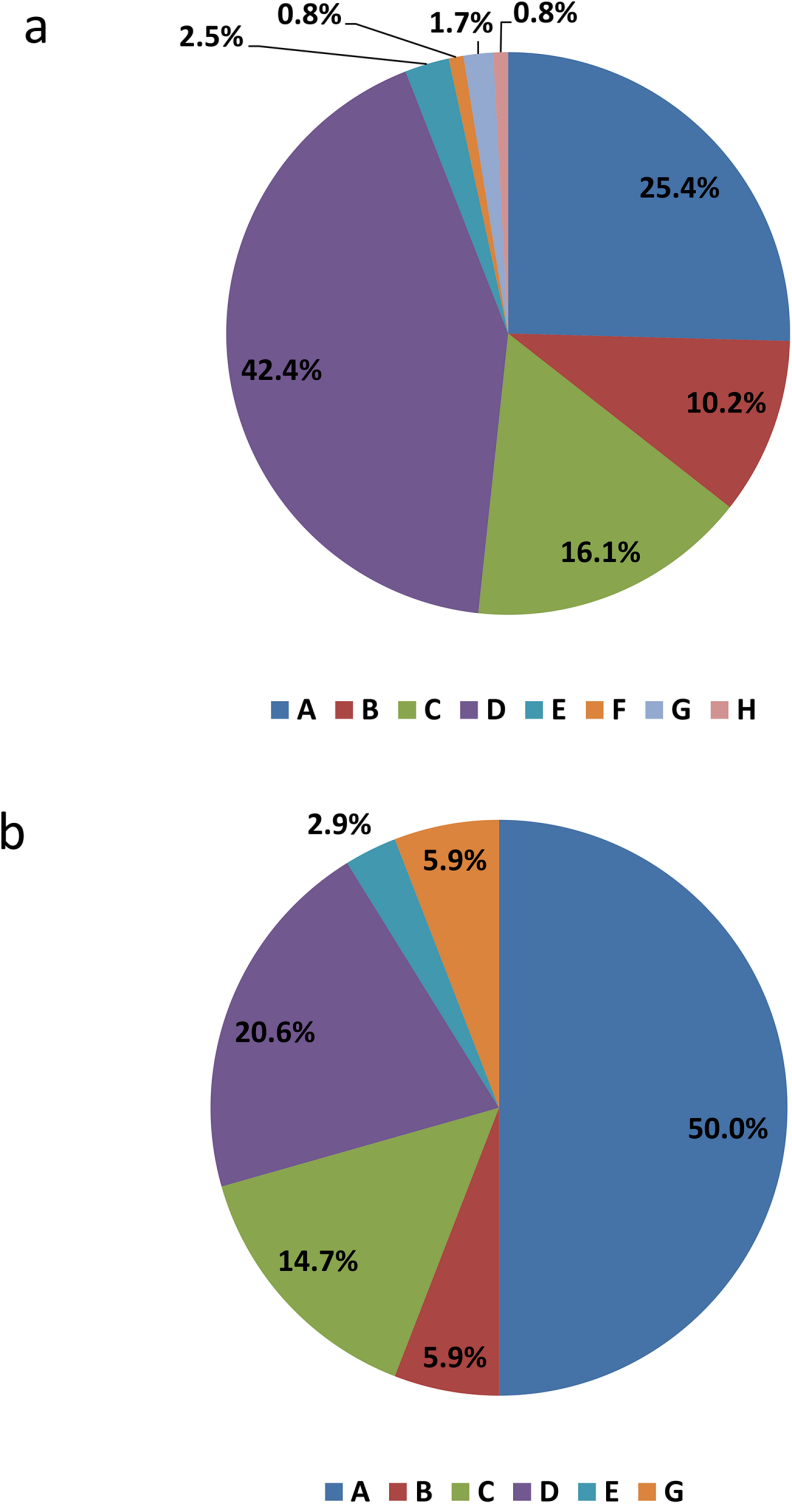
Genotype distribution for acute HBV cases from Western Canada (n = 118; a), and Central Canada (n = 34; b).

**Fig 3:**
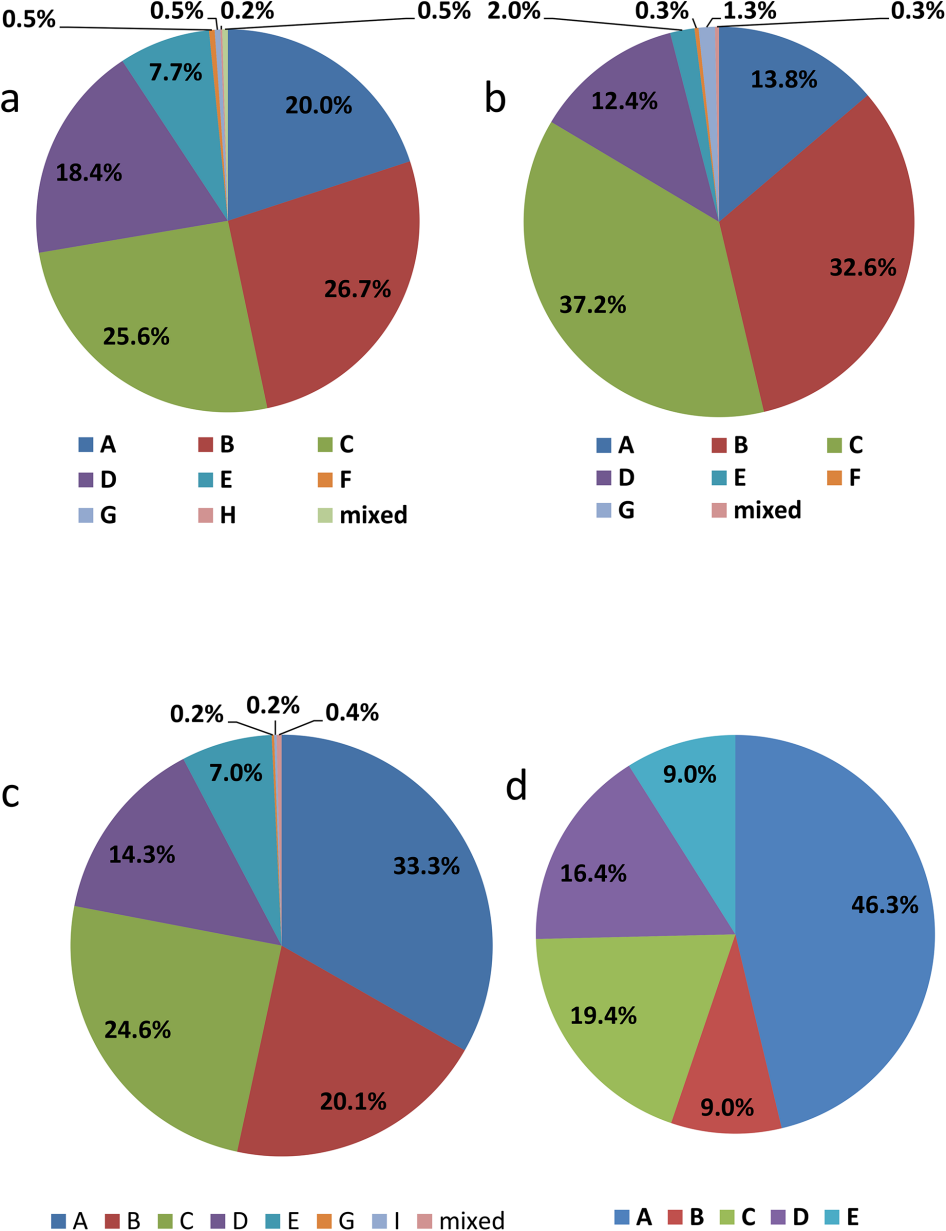
Genotype distribution for chronic HBV cases from Western Canada (n = 636; a), Central Canada (n = 298; b), the Prairies region (n = 532; c), and the Maritimes region (n = 67; d).

The relative distribution of acute and prevalent HBV cases by genotype is shown in Table 1. The relative distribution of acute and prevalent HBV cases by genotype, year of report and reporting region is shown in Figs 1–3.

**Table 1:** Relative genotype distribution of acute and chronic HBV cases.

Genotype (n)	Acute HBV cases		Prevalent HBV cases		Fisher’s exact p-value for comparing proportions
	N = 152	Percentage (95%CI)	N = 1533	Percentage (95%CI)	
**A**	47	30.9% (24.0–38.8)	376	24.5% (22.4–26.7)	0.095
**B**	14	9.2% (5.5–15.0)	380	24.8% (22.7–27.0)	<0.001
**B/C**	0	n/a	3	0.2% (0.1–0.6)	1.0
**C**	24	15.8% (10.8–22.6)	418	27.3% (25.1–29.6)	0.002
**C/D**	0	n/a	3	0.2% (0.1–0.6)	1.0
**D**	57	37.5% (30.1–45.5)	241	15.7% (14.0–17.6)	<0.001
**E**	4	2.6% (1.0–6.9)	98	6.4% (5.3–7.7)	0.078
**F**	1	0.7% (0.1–4.6)	4	0.3% (0.1–0.7)	0.377
**G**	4	2.6% (1.0–6.9)	8	0.5% (0.3–1.0)	0.018
**H**	1	0.7% (0.1–4.6)	1	0.1% (0.01–0.5)	0.172
**I**	0	n/a	1	0.1% (0.01–0.5)	1.0

### Demographic characteristics

Among acute HBV samples, 70% were from male subjects, and among chronic HBV cases, only 53% were from males (Fisher’s exact test p<0.001).

The mean age of acute HBV cases was 43.2 (95%CI: 40.8–45.6) and the mean age of chronic HBV cases was 40.6 years (95%CI: 39.8–41.5) with the difference in means being not significant by t-test (p = 0.07).

### Genotype

#### Acute HBV

**Fig 4:**
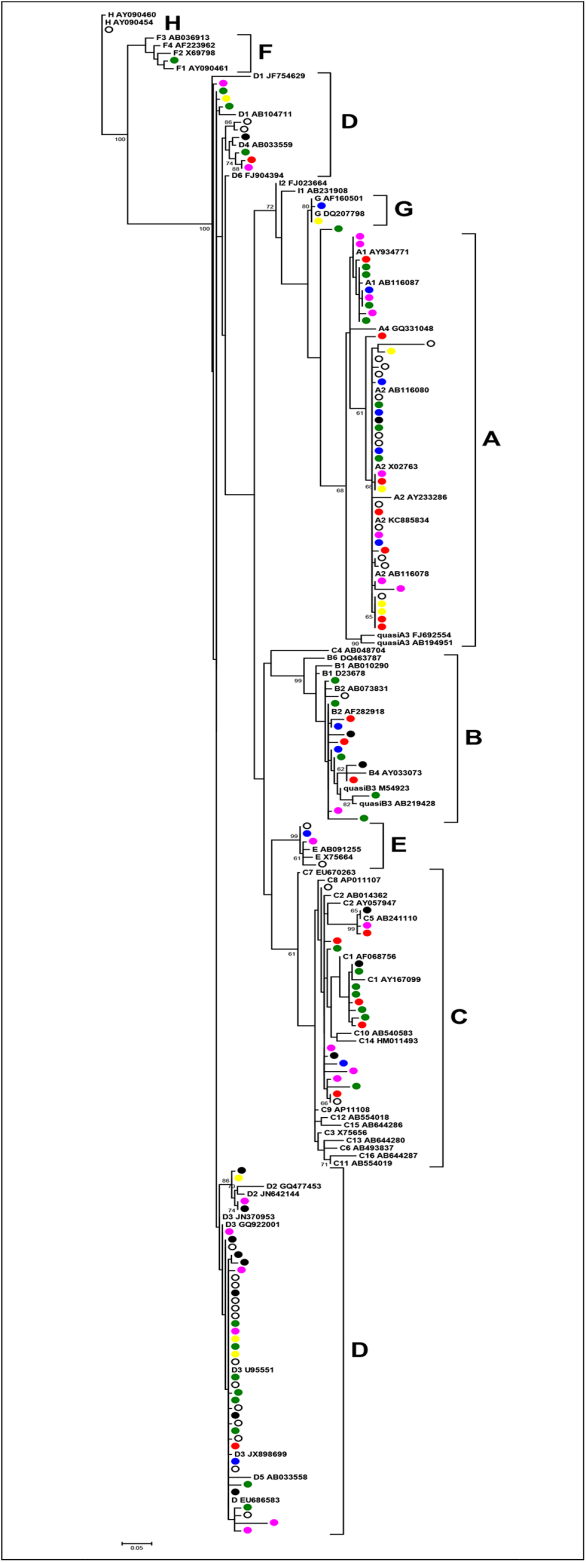
Phylogenetic analysis of Canadian acute case HBV sequences from 2006 to 2012. An alignment of 139 acute case sequences having sufficient length (519 bp; nucleotide 311 to 829) together with GenBank reference sequences were analyzed by maximum likelihood analysis using DIVEIN software with tree construction by the BioNJ algorithm with 100 bootstrap replicates. Acute case sequences are shown as circles, by year; 2006 (black filled circle), 2007 (blue filled circle), 2008 (open circle), 2009 (green filled circle), 2010 (pink filled circle), 2011 (red filled circle), 2012 (yellow filled circle). GenBank sequences are designated by the HBV genotype or subgenotype followed by the accession number. Bootstrap confidence values of ≥60% are given. The ruler shows the branch length for a pairwise distance equal to 0.05.

The partial HBsAg-coding region of sufficient length (approximately 519 bp from nucleotide 311 to 829) was available for phylogenetic analysis of 139 specimens collected over the 7-year period from acutely infected individuals. Clustering among different isolates of a particular year was not observed, although most genotype A and genotype D sequences shared complete identity, respectively (Fig 4), suggesting the possibility of persistently circulating genotype A and D strains from year to year.

**Fig 5:**
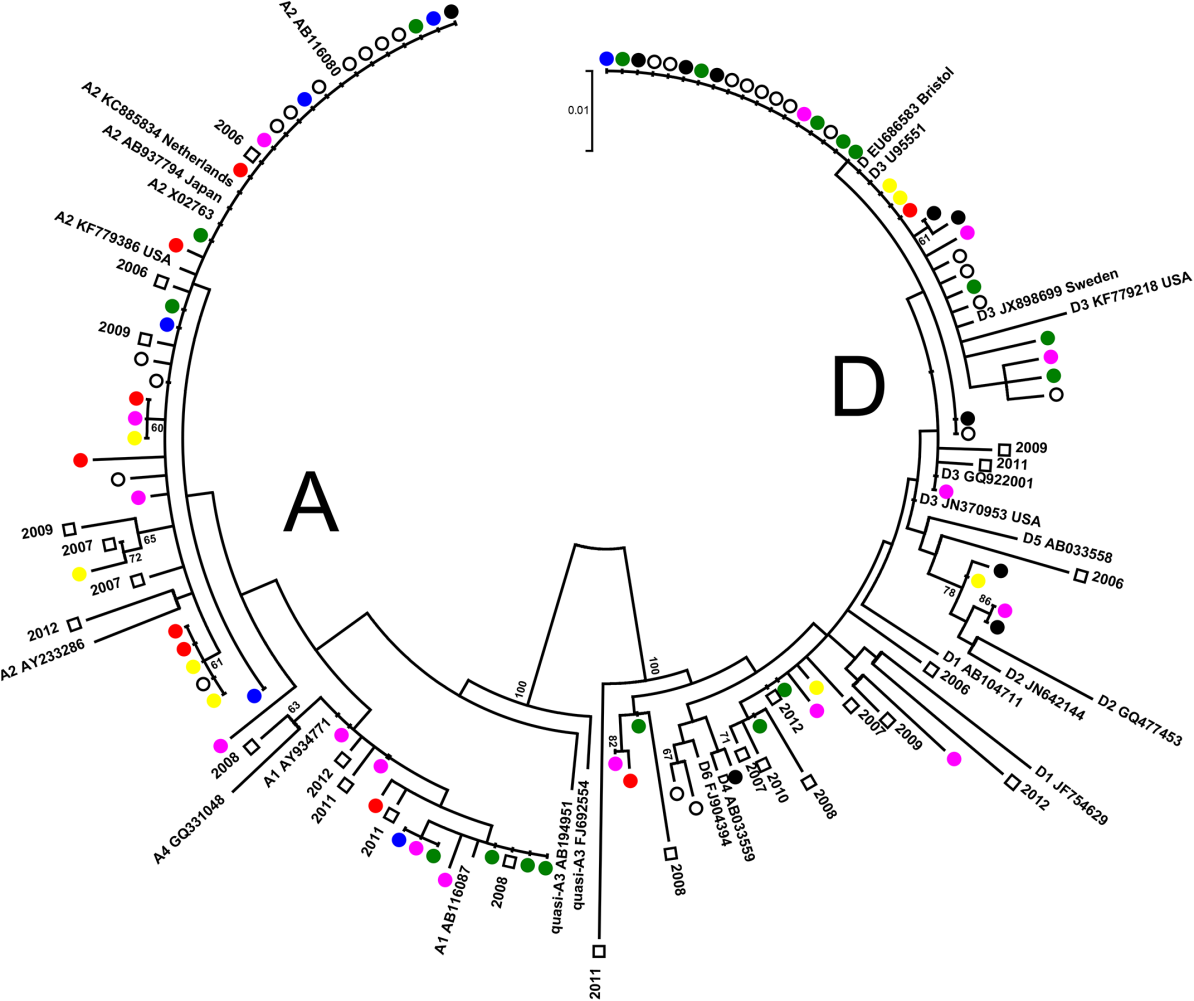
Phylogenetic analysis of Canadian HBV acute and prevalent case genotype A and D sequences from 2006 to 2012. An alignment of 50 genotype D and 43 genotype A sequences having sufficient length (519 bp; nucleotide 311 to 829) together with GenBank reference sequences and sequences from the prevalent population within the same regions as acute cases (2 per year per genotype, except 2010, with 1 for genotype D for the same geographic region, due to almost exclusive INNO-LiPA analysis for reference requests in 2010) were analyzed by maximum likelihood analysis using MEGA5.2 software with 500 bootstrap replicates. Acute case sequences are shown as circles, by year; 2006 (black filled circle), 2007 (blue filled circle), 2008 (open circle), 2009 (green filled circle), 2010 (pink filled circle), 2011 (red filled circle), 2012 (yellow filled circle). Comparative prevalent population sequences are shown as squares together with the year. GenBank sequences are designated by the HBV subgenotype followed by the accession number and country or city, if referring to an acute case reference sequence. Bootstrap confidence values of ≥60% are given. The ruler shows the branch length for a pairwise distance equal to 0.01.

To determine if genotype A and D strains from acutely infected individuals shared identity with strains from the prevalent population, phylogenetic analysis was performed with HBsAg sequence from both acutely- and chronically- infected persons within the same geographic regions. Genotype A (N = 43) and D (N = 50) HBsAg sequences from acute cases were analyzed together with genotype A and genotype D prevalent population sequences (Fig 5). No specific clustering between chronic and acute sequences in any given year was observed for either genotype.

#### Prevalent HBV

A total of 693 specimens were genotyped by INNO-LiPA, while 840 were genotyped by sequence analysis. The partial HBsAg-coding region sequence of sufficient length (approximately 519 bp from nucleotide 311 to 829) was available from a selection (n = 460) of prevalent cases for construction of a phylogenetic tree, with the remaining sequence data either not available following archival loss (n = 35) or not of sufficient length (n = 345). Phylogenetic analysis demonstrated clustering of sequences within each genotype clade with >70% bootstrap value, but no specific clustering of specimens within a year or from year to year was observed with strong bootstrap support (S1 Fig).

### Genomic Characterization

#### Acute HBV

Amino acid substitutions within the HBsAg/partial polymerase overlapping region and BCP/PC region were investigated with 146 and 139 sequences, respectively. Most polymerase sequences from acutely-infected individuals lacked substitutions associated with drug resistance, except for 4 (2.7%) specimens (rtL180M/rtM204V –genotype G; rtL180M/rtM204V/rtV173L –genotype A; rtM250M/L–genotype A; rtA181A/S–genotype B). Similarly, substitutions in HBsAg sequences associated with immune escape were infrequently observed. One sequence (genotype B) had 3 mutations associated with immune escape; K141I, D144A, and G145R.

Genotype D sequences among acute cases were highly characterized by a methionine at amino acid 125 within the HBsAg antigenic determinant. Of the 50 genotype D sequences having partial HBsAg-coding region sequence available, 32 (64%) had M125 while 18 (36%) had T125. This contrasted with genotype D prevalent case sequences, which were overwhelmingly T125 (64/69; 93%).

Following sequence analysis to investigate BCP/PC mutation prevalence, the PC stop codon mutation A1896 was observed with 20 (14%) sequences; 5 genotype B, 4 genotype C, 10 genotype D and 1 genotype E. Mutations within the BCP were observed with 25 (18%) sequences, including 17 sequences having the T1762/A1764 mutation (4 genotype A, 2 genotype B, 5 each genotype C and D, 1 genotype E), 4 sequences having the A1762/A/T1764 mutation (1 each genotype A, B, C, and D), and 4 sequences having deletions (3 genotype C) or an insertion (1 genotype A) within a region encompassing nt 1755 to nt 1777.

#### Prevalent HBV

Amino acid substitutions within the HBsAg-coding and partial polymerase-coding region were investigated in the 460 sequences used for phylogenetic analysis from HBV prevalent patients. Mutations associated with vaccine or diagnostic escape [21, 22] were observed among samples from all genotypes, except genotypes F and H, and included HBsAg substitutions at G119, P120, T123, I/T126, Q129, M133, S136, S/T140, P142, D144, G145, E164, and I195 (19% of sequences). Mutations in the polymerase-coding region leading to drug resistance (rtI169T, rtV173L, rtL180M, rtA181V/T, rtT184I/S, rtV191I, rtA194T, rtM204V/I), were observed mainly in genotypes A to E and G (24% of sequences), due to many samples being from nucleos(t)ide analogue experienced patients tested for treatment management.

### Demographic and risk characteristic analyses

#### Acute HBV

Table 2 provides univariate and multivariate analyses for genotypes A, B, C and D, presented as odds ratios from logistic regression models. In multivariate analyses, history of exposure to HCV (determined as positive anti-HCV test result) was positively associated with genotype D, while year of diagnosis and being HBeAg positive was positively associated with genotype A.

**Table 2:** Adjusted and unadjusted odds ratios from single-factor and multiple logistic regression analyses for HBV genotypes A, B, C and D.

Variable	Genotype A				Genotype B				Genotype C				Genotype D			
	Acute HBV cases		Chronic HBV cases		Acute HBV cases		Chronic HBV cases		Acute HBV cases		Chronic HBV cases		Acute HBV cases		Chronic HBV cases	
	Odds ratio (95%CI)	Adjusted odds ratio (95%CI)	Odds ratio (95%CI)	Adjusted odds ratio (95%CI)	Odds ratio (95%CI)	Adjusted odds ratio (95%CI)	Odds ratio (95%CI)	Adjusted odds ratio (95%CI)	Odds ratio (95%CI)	Adjusted odds ratio (95%CI)	Odds ratio (95%CI)	Adjusted odds ratio (95%CI)	Odds ratio (95%CI)	Adjusted odds ratio (95%CI)	Odds ratio (95%CI)	Adjusted odds ratio (95%CI)
Year of diagnosis^a^	1.3 (1.0–1.6)^b^	1.4 (1.1–1.8) ^b^	1.1 (1.1–1.2)^b^	1.1 (1.0–1.2)^b^	0.9 (0.6–1.2)		1.0 (0.9–1.0)		1.1 (0.8–1.3)		0.9 (0.8–0.9)^b^	0.9 (0.8–1.0)^b^	0.8 (0.7–1.0)^b^	0.8 (0.6–1.0)^b^	1.1 (1.0–1.2)^b^	1.1 (0.9–1.2)
Being female	0.5 (0.1–1.1)	0.3 (0.1–0.8)^b^	0.8 (0.6–1.0)	0.9 (0.7–1.1)	0.5 (0.1–4.1)		1.3 (1.0–1.7)^b^	1.3 (1.0–1.7)^b^	2.3 (0.8–6.0)		1.5 (1.1–1.9)^b^	1.4 (1.1–1.9)^b^	1.5 (0.7–3.2)		0.7 (0.5–0.9)^b^	0.6 (0.5–0.9)^b^
Being aged 41+	1.7 (0.8–3.6)		1.0 (0.8–1.3)	1.0 (0.8–1.3)	5.2 (0.6–45.6)		1.6 (1.2–2.1)^b^	1.6 (1.2–2.0)^b^	0.6 (0.2–1.6)		1.3 (1.0–1.6)		0.8 (0.4–1.6)		0.7 (0.5–0.9)^b^	0.7 (0.5–0.9)^b^
History of having received HBV treatment or HBV vaccine or been tested for STBBI	1.8 (0.8–4.0)				5.7 (1.0–32.9)	11.1 (1.0–120.3)^b^			0.4 (0.1–1.5)				0.4 (0.2–1.0)^b^			
History of having received repeated STBBI testing	2.7 (1.1–6.7)^b^				5.0 (0.9–26.6)				0.2 (0.0–1.6)^b^	0.1 (0.0–0.9)^b^			0.6 (0.2–1.7)			
Being HBeAg positive	3.7 (1.5–9.0)^b^	4.2 (1.3–13.5)^b^			0.8 (0.1–6.8)				0.6 (0.2–2.4)				0.4 (0.1–1.1)			
Being anti-HCV positive	0.2 (0.1–1.1)	0.2 (0.1–1.0)^b^			1				0.3 (0.0–2.4)				6.6 (2.0–21.7)^b^	6.8 (2.0–22.9)^b^		
History of immigration	0.5 (0.2–1.2)	0.3 (0.1–0.7)^b^			1.0 (0.2–5.9)				1.5 (0.6–3.9)				1.0 (0.5–2.2)			
Being of Asian ethnicity	0.5 (0.1–1.8)				9.1 (1.4–60.4)^b^	12.5 (1.6–98.8)^b^			12.6 (3.5–44.6)^b^	19.0 (4.3–83.8)^b^			1			
Reporting clinical symptoms of being unwell	1.0 (0.4–2.3)				1.8 (0.2–16.2)				1.5 (0.5–4.9)				0.6 (0.3–1.3)			
ALT>2.5 times the upper normal limit	0.7 (0.1–4.6)				1				0.7 (0.1–7.0)				2.6 (0.3–24.2)			
History of invasive medical procedures (life-time)	0.8 (0.4–1.8)				1.4 (0.3–7.5)				0.9 (0.4–2.5)				1.0 (0.5–2.0)			
Residing with HBV carrier or IDU (life-time)	0.7 (0.3–1.6)				1.0 (0.2–5.7)				1.4 (0.5–3.8)				1.1 (0.5–2.4)			
History of sexual intercourse with HBV-infected person or men who have sex with men (MSM) (life-time)	1.32 (0.5–3.2)				0.8 (0.1–6.8)				1.8 (0.6–5.4)				0.3 (0.1–0.9)^b^			
History of unprotected heterosexual intercourse (life-time)	0.9 (0.4–1.8)				0.4 (0.1–2.5)				1.4 (0.5–3.8)				1.2 (0.6–2.5)			
History of incarceration (life-time)	1.2 (0.4–3.3)				1				0.3 (0.0–2.0)				1.5 (0.6–4.0)			
History of occupational exposure (life-time)	0.9 (0.4–2.3)				0.7 (0.1–6.2)				0.3 (0.1–1.6)				1.8 (0.8–4.2)			
History of IDU (life-time)	1.0 (0.4–2.9)				1				0.3 (0.0–2.2)				2.2 (0.8–6.1)			
History of receiving tattoo or piercing (life-time)	0.8 (0.3–1.8)				1.2 (0.2–6.9)				0.8 (0.3–2.3)				1.1 (0.5–2.4)			
Having >1 exposure known to cause HBV infection (life-time)	1.1 (0.5–2.3)				0.6 (0.1–2.9)				1.4 (0.5–4.0)				0.9 (0.4–1.8)			

^a^diagnosis for Acute HBV cases/testing for Chronic HBV cases.

^b^indicates p<0.05 (All Fisher tests used double-sided p-values).

Regardless of their hepatitis B genotype, those reporting multiple (i.e. > 1) risk factors were more likely to report any or liver-specific clinical symptoms of their liver disease (Fisher’s exact test p<0.001 for each). Those reporting multiple exposures were almost twice more likely to report more contact with healthcare services in the past, such as having received HBV treatment, HBV vaccine or STBBI testing (Pearson chi-square p = 0.048).

After we’ve applied an algorithm to define an individual’s single likely exposure based on the probability of HBV transmission in a mutually exclusive and deterministic way that is described in the footnote to Table 3, history of incarceration (24.6%; 95%CI: 17.8–33.0) and residing with a chronic HBV carrier or injection drug user (23.0%; 95%CI: 16.4–31.3) were found to be the top two risk factors. The complete relative distribution of risk factors associated with acute HBV is presented in Table 3 and, except for reported heterosexual exposure category, demonstrates a good fit with US data [23].

**Table 3:** Complete relative distribution of risk factors associated with acute HBV.^a^

Exposure	Proportion % (95%CI) N = 126	US data (% estimate or range)
Prison	24.6 (17.8–33.0)	13–47
Residence with a chronic HBV carrier or IDU	23.0 (16.4–31.3)	14–60
Sexual intercourse with IDU or MSM	18.3 (12.4–26.1)	18 (MSM only)
History of Injection drug use	14.3 (9.1–21.7)	16
Unprotected heterosexual intercourse	14.3 (9.1–21.7)	32
Immigration	4.8 (2.1–10.3)	?
Occupational exposure	0.8 (0.1–5.6)	?

^a^Defining has been carried out in a pre-determined and re-iterative way: First, all exposures were ranked according to their potential to cause an acute HBV by a measure of transmission risk and the likely duration of exposure as follows: High = IDU, Sex with HBV and MSM, having a household HBV carrier or IDU, heterosexual exposure; Medium-High = Immigration, prison history, occupational exposure; Medium-low = tattoo or piercing; low = invasive medical procedures or non-injection drug use.

#### Prevalent HBV

In multivariate analyses (Table 2), female sex was positively associated with genotypes B and C and negatively associated with genotype D. Older age (41+) was positively associated with genotype B and negatively associated with genotype D. Genotype A was positively associated and genotype C was negatively associated with the year of specimen testing.

## Discussion

The current study details for the first time the annual and regional genotype distribution of both prevalent and acute infection throughout Canada in a relatively large set of samples collected between 2006 and 2012. The results show differences in how HBV genotypes are distributed among individuals acutely and chronically infected with HBV and describe associations with known risk factors, suggesting differences in the transmission risk and potential disease outcomes, and offers an insight into a more targeted prevention.

HBV is a nationally notifiable disease in Canada [24]; however, until recently, reporting has not differentiated between acute and chronic cases [25], and infection status has not been reported consistently across the country due to misclassification and delayed reporting [25]. Also, because of the largely asymptomatic nature of HBV infection and existing differences in access to testing across Canadian populations, both acute and chronic HBV are likely underreported in Canada [1].

Similar to findings from other developed countries [26–28], reported case rates of acute HBV in Canada appear to be declining over time [8, 25, 29]. Even so, acute infection persists, with rates of reported cases among men twice as high as in women, with age-specific patterns observed; the highest rates in men were in ages 35–54, compared to the highest rates in females, which were in individuals aged 25–34 years.

Although serological screening for chronic hepatitis B is not routinely conducted in immigrants entering Canada [3, 30], in light of evidence suggesting an estimated 58% to 72% of all HBsAg carriers to be first-generation immigrants [31] and that the majority of chronically-infected Canadians are born abroad [1], it is suspected that continuous immigration from HBV endemic areas is contributing to a flattened or possibly rising prevalence of chronic hepatitis B in Canada [25, 32].

In our study, acute infection among individuals younger than 18 was not observed, likely due to implementation of HBV vaccination programs starting in the mid-1990’s throughout Canada [33–35] and evidence of higher population measures of vaccine-induced immunity in younger individuals [1]. In contrast, the age range of the prevalent sub-sample population in this study included infants aged less than 1 year old to seniors aged 96, likely reflecting different risk factors involved and clinical pathways (i.e. with and without clinically acute hepatitis) associated with chronic HBV infection in the Canadian population [36].

Acutely infected individuals infected with genotype A were more likely to be Canadian-born and less likely to have a history of exposure to HCV while those infected with genotype D were more likely to have been exposed to HCV, which in our view, may be a more objective proxy measure for the self-reported history of injection drug use in light of the evidence of a sizeable proportion of injectors becoming HCV infected within 2 years from initiating [37] and the considerable social stigma attached to this practice [38].

In multivariate analyses, both genotypes B and C demonstrated strong positive associations with reported Asian heritage, but were at odds in associations with proxy measures for accessing healthcare (e.g. STBBI testing, hepatitis treatment or vaccination), with genotype B carriers being more likely to have a history of accessing health care and genotype C carriers being less likely to do so. While reporting accessing healthcare services in those with genotype B was positively associated with having clinical symptoms of liver disease (p = 0.008) and negatively with having ALT 2.5 times above the normal limit (p = 0.057), this finding warrants further examination given that genotype C has been associated with poorer clinical prognosis than genotype B despite lower viral load and more favorable HBeAg status [39]. In addition, access to care depends on a number of individual and structural factors, including gender, local language proficiency, beliefs, income and hours of work, as well as proximity and availability of health services [31, 40], none of which were explored in our analysis.

The majority of acute HBV cases (63.5%; 95%CI: 54.6–71.5) reported multiple exposures commonly known to be associated with HBV infection. After we’ve applied the probability based algorithm to determine a single likely exposure, history of incarceration and residing with a chronic HBV carrier or IDU were identified as the two most likely risk factors. This finding may be due to more pro-active health-seeking behavior of this group (determined as history of repeated testing for STBBI, HBV treatment or HBV vaccination) and willingness to disclose risk factor information, which were found positively associated by Pearson chi-square test with p = 0.048.

Many countries investigating HBV genotype prevalence within the incident population [41–43], have observed a shift away from prevalent circulating genotypes to “foreign”-derived genotypes, such as an observed shift from genotype C to A in Japan [44, 45]. As chronic hepatitis B in Canada is believed to largely affect immigrants from endemic regions of the world [5], it might be expected that genotypes found within the chronic population would form the circulating strains within the incident population. The present study demonstrated a division between genotype strains predominant in these two populations. Genotypes A and D jointly comprised 68.4% (95%CI: 60.5–75.4) of all acute infections, yet only 40.2% (95%CI: 37.8–42.7) of infections in the prevalent population. While in multivariate analyses, genotype A demonstrated an increasing trend within acute and chronic cases over time, there was evidence of decreases in the proportions of genotype C among chronic HBV cases and genotype D among acute HBV cases (Table 2). The observed increasing trend of genotype A within the Canadian HBV infected population may have unwanted consequences due to the association of genotype A infection with persistent viremia [46], despite low replicative activity [47], along with a higher risk of developing chronic infection [45, 48, 49], suggesting escape of host immune responses compared to other HBV genotypes.

Although subgenotype analysis could not be adequately performed on subgenomic regions, it was found that the majority of genotype A acute samples having available sequence for amino acids 207 and 209 (32/43; 74%) had HBsAg-coding region amino acids characteristic of subgenotype A2 (S207, V209), while the majority (79/112; 71%) of genotype A prevalent samples having sufficient sequence data had amino acids characteristic of subgenotype A1 (N207, L209 [50]). Similarly, the majority of acute HBV/D cases (32/50; 64%) with sufficient sequence information had a methionine residue at amino acid 125 while 18 (36%) had T125, which contrasted with genotype D prevalent case sequences, which were overwhelmingly T125 (64/69; 93%). M125 HBsAg sequences are a signature for subgenotype D3 specimens associated with injection drug use [51]. The difference in amino acid 125 specificity between the incident and prevalent sequences further substantiates the strain difference observed between the two populations.

Many of the HBV/A acute sequences were identical to the previously described “prisoner variant” (HBV^PV^), associated with incarceration, IDU, or MSM throughout England and the Netherlands [52, 53]. Similarly, the majority of HBV/D acute sequences shared sequence identity with a well-known variant, HBV^BV^, circulating for decades in Europe (“Bristol” strain), also associated with outbreaks of infection among injection drug users [51, 54]. Interestingly, mutations within the precore (G1896A) or basal core promoter (A1762T/G1764A) regions of the HBV genome leading to reduction or loss of HBeAg, were observed in a low percentage (<20%) of acute case sequences. Mutations in both regions have been associated with fulminant hepatitis during primary infection [55].

An important detail resulting from recurrent sequence identity among disparate acute cases is the lack of epidemiological causality and relationship, despite close genetic relatedness. Thus, although most genotype A and genotype D sequences shared complete identity, respectively, based on phylogenetic analysis (Figs 4 and 5), caution is required when estimating genetic and transmission relatedness based only on HBsAg or subgenomic sequences [46]. Ultimately, whole genome sequencing and/or next generation sequencing will be required to evaluate the genetic relatedness of sequences associated with acute infection.

This study has several limitations. Demographic and epidemiological data was not available for all specimens, which may have affected our ability to detect associations with known risk factors. Also, the reported regional distribution of genotypes, in our view, reflects established reporting practices rather than actual genotype distribution in the populations within geographically defined boundaries. The conclusions based on newly diagnosed cases from the participating regions may not be representative of the whole country. Similarly, the results from the prevalent population involving referral specimens may be subject to a selection bias and so may not represent all Canadian HBV patients.

In summary, monitoring of HBV genotypes in the Canadian population has demonstrated the impact that immigration patterns have had on chronic HBV genotype prevalence throughout the country, and how this differs from currently circulating incident strains. Linkage of HBV genotype with specific risk factors helps identify potential clusters of transmission, and may point to possible gaps in preventative measures among these risk groups. Finally, molecular epidemiological surveillance provides a measure of the stability and expansion of circulating strains [46], which together with an understanding of genotype prevalence and distribution may help in implementing appropriate measures for prevention and treatment. Increased surveillance involving whole genome sequence will be essential for full variant characterization and enhanced epidemiological interpretations.

## Supporting Information

S1 Fig Phylogenetic analysis of Canadian prevalent case HBV sequences from 2006 to 2012. An alignment of 460 prevalent case sequences having sufficient length (519 bp; nucleotide 311 to 829) together with GenBank reference sequences were analyzed by maximum likelihood analysis using DIVEIN software with tree construction by the BioNJ algorithm with 100 bootstrap replicates. Prevalent case sequences are shown as circles, by year; 2006 (black filled circle), 2007 (blue filled circle), 2008 (open circle), 2009 (green filled circle), 2010 (pink filled circle), 2011 (red filled circle), 2012 (yellow filled circle). GenBank sequences are designated by the HBV genotype or subgenotype followed by the accession number. Bootstrap confidence values of ≥60% are given. The ruler shows the branch length for a pairwise distance equal to 0.02.
(TIFF)

S1 Table Raw data for Canadian acute HBV cases from 2006 to 2012. (XLSX)

S2 Table Raw data for Canadian prevalent HBV cases from 2006 to 2012. (XLSX)
